# On the open-source landscape of *PLOS Computational Biology*

**DOI:** 10.1371/journal.pcbi.1008725

**Published:** 2021-02-11

**Authors:** Mathieu Boudreau, Jean-Baptiste Poline, Pierre Bellec, Nikola Stikov

**Affiliations:** 1 Montreal Heart Institute, Montréal, Québec, Canada; 2 NeuroPoly Lab, Polytechnique Montréal, Montréal, Québec, Canada; 3 Montreal Neurological Institute, McGill University, Montréal, Québec, Canada; 4 Centre de recherche de l’Institut universitaire de gériatrie de Montréal, Montréal, Québec, Canada; 5 Université de Montréal, Montréal, Québec, Canada

Over the past year, I (M.B.) have been investigating the landscape of code-sharing in academic journals across different research fields. At the end of my PhD, I made the choice to share code that reproduces figures from one of my papers [1], and since then, I’ve been involved in several open-source projects (qMRLab and AxonDeepSeg) and initiatives dealing with open science in publishing (NeuroLibre and Canadian Open Neuroscience Platform). Recently, following an editorial by N.S. on reproducibility and the future of MRI research [2], we wrote a blog post presenting an analysis of the open-source landscape for the journal *Magnetic Resonance in Medicine* (MRM), which broadly focuses on MRI research for medical applications. These findings provided a snapshot of the current state of the open-source landscape for that journal (e.g., most used coding language is still MATLAB) and some insights into new trends (12% of the articles shared code that reproduced figures). In this editorial, we examine the open-source landscape of *PLOS Computational Biology*. *PLOS Computational Biology* is inherently different from MRM not only because of the difference in research topics, but also because it’s an open-access journal that focuses primarily on computational studies.

The broad questions that were of interest are the following:

- What percentage of *PLOS Computational Biology* publications claim to share code?
- If they share code, what coding languages do they use?
- Where do the authors typically host their code?
- How many publications share scripts that reproduce some or all of the figures from their paper?

## Details of analysis

To perform this analysis, all the articles published in *PLOS Computational Biology* from January to December 2019 were downloaded. A script was then executed to search for all the articles that contained one of a list of keywords that may hint at containing code/data. Following that, all the articles that matched keywords were compiled into a Google Sheet file and manually searched inside each of those articles to determine if the code they used was actually shared. The external links in the articles were then examined to see if they (1) shared code; (2) see which languages the code used; (3) where they hosted their code; and (4) if the code aimed to reproduce any of the figures. See Table 1 for an overview of results.

**Table 1:** Broad overview of results.

**Total number of articles (Jan to Dec 2019)**	622
**# of articles that matches at least 1 keyword**	440 (71%)
**# of articles that actually linked to shared code**	255 (41%)
**Most common coding language**	Python (45%)
**Most common location where code is hosted**	GitHub (75%)

Overall, 41% of the articles published in *PLOS Computational Biology* reported sharing some code. It is possible that the rate is even slightly higher, as some articles that reported sharing code may not have used one of the keywords in the search set I used. Initially, we also aimed to look at comparing how many articles shared their data as well. However, it was too challenging to differentiate between articles that *shared* data with articles that *used* shared data, as this distinction was not always clear in the data availability statement.

Python was the most commonly used programming language for *PLOS Computational Biology*; 45% of articles that shared code used Python. MATLAB was the second most common programming language (30%). Using open-source languages like Python opens the gateway to a wide selection of other open-source tools (e.g., continuous integration, Jupyter Notebook, Binder, etc.) which are mostly incompatible with licensed software like MATLAB. However, people are likely to choose to continue to code in the language they are most comfortable with, and they will likely stick with the coding language mostly used in their research labs, which may be part of the reason why MATLAB is still widely used even for this journal.

Shared code was mostly hosted on GitHub (75%). Beyond simply sharing code, some articles also used some state-of-the-art tools for sharing reproducible research. These include sharing Jupyter Notebooks, Docker images or Dockerfiles, and MyBinder links. Overall, of the articles that shared code, the percentages of those that also used these reproducibility tools are shown in Table 2.

**Table 2:** Reproducibility tools used by percentage.

**Shared Jupyter Notebooks**	17%
**Used Docker/Dockerfiles**	3%
**Shared a MyBinder link**	1.2%
**Had scripts that claimed to reproduce some or all figures**	11%

In addition to sharing code, an emerging trend in the open science community is to provide an easily reproducible coding environment that requires only a web browser to run demos or reproduce figures. Several tools and services are available to do this, such as MyBinder, Google Colab, NeuroLibre, and Code Ocean. In addition, sharing interactive figures (e.g., Plotly and Bokeh), widgets (e.g., ipywidget), or dashboards (e.g., Dash and Shiny) can further enhance interactivity with code developed for the paper, and provide an additional tool for the reader to deepen their understanding of the work. There may exist a steep learning curve to create these interactive documents, as some knowledge is required (i.e., Docker and environment configuration files). However, the required skills can be developed within labs in a short time frame. As an example, we took two recently published papers in *PLOS Computational Biology* [3,4] and customized the code provided by the authors to create NeuroLibre-style Jupyter Books with interactive figures using Plotly (Example 1 and Example 2). As part of an experimental collaboration between NeuroLibre and *PLOS Computational Biology*, the journal team is seeking user feedback on these examples to further understand the value of these features to the journal’s community.

Overall, *PLOS Computational Biology* appears to have a user base that has embraced the culture of sharing code. Not only do many publications share code, but they are also using coding languages that are open source. A sizable portion of them are using tools that are compatible with these open-source coding languages (e.g., Python), such as various Project Jupyter initiatives (Jupyter Notebooks and MyBinder) and dockerizing their projects. However, only a small number of articles used their code to share scripts that reproduce figures (about 1 in 10), meaning that there is still some progress to be made in terms of sharing reproducible research. We’re anticipating redoing this analysis in a year or two, to see how some of these statistics have evolved over time. Our sincere hope is that by then, reproducibility takes center stage in the evolving landscape of academic publishing.
